# Continuous Glucose Monitoring in the Management of Neonates With Persistent Hypoglycemia and Congenital Hyperinsulinism

**DOI:** 10.1210/clinem/dgab601

**Published:** 2021-08-18

**Authors:** Myat Win, Rowan Beckett, Lynn Thomson, Ajay Thankamony, Kathryn Beardsall

**Affiliations:** 1 Department of Paediatrics, Cambridge University Hospitals NHS Foundation Trust, Cambridge CB2 0QQ, UK; 2 School of Clinical Medicine, University of Cambridge, Cambridge CB2 0SP, UK; 3 Department of Paediatrics, University of Cambridge, Cambridge CB2 0QQ, UK

**Keywords:** Glucose, hyperinsulinism, hypoglycemia, continuous glucose monitoring

## Abstract

**Background:**

Persistent hypoglycemia is common in the newborn and is associated with poor neurodevelopmental outcome. Adequate monitoring is critical in prevention, but is dependent on frequent, often hourly blood sampling. Continuous glucose monitoring (CGM) is increasingly being used in children with type 1 diabetes mellitus, but use in neonatology remains limited. We aimed to introduce real-time CGM to provide insights into patterns of dysglycemia and to support the management of persistent neonatal hypoglycemia.

**Methods:**

This is a single-center retrospective study of real-time CGM use over a 4-year period in babies with persistent hypoglycemia.

**Results:**

CGMs were inserted in 14 babies: 8 term and 6 preterm infants, 9 with evidence of congenital hyperinsulinism (CHI). A total of 224 days of data was collected demonstrating marked fluctuations in glucose levels in babies with CHI, with a higher sensor glucose SD (1.52 ± 0.79 mmol/L vs 0.77 ± 0.22 mmol/L) in infants with CHI compared with preterm infants. A total of 1254 paired glucose values (CGM and blood) were compared and gave a mean absolute relative difference of 11%.

**Conclusion:**

CGM highlighted the challenges of preventing hypoglycemia in these babies when using intermittent blood glucose levels alone, and the potential application of CGM as an adjunct to clinical care.

Hypoglycemia is a common problem in the neonatal period but is usually transient (1). Persistent and severe neonatal hypoglycemia is typically found in preterm or growth-restricted babies who cannot tolerate fasting, or in infants with congenital hyperinsulinism (CHI). Persistent hypoglycemia can lead to poor neurobehavioral outcomes in childhood, with up to 48% of children with CHI having neurodevelopmental deficits (2, 3). Advances in understanding molecular mechanisms and development of drug treatments such as diazoxide and chlorothiazide for CHI have led to first-line management of many babies now being undertaken in local neonatal units (4). Local unit management requires frequent blood sampling to ensure normoglycemia while weaning off IV dextrose support. Current recommendations advise obtaining hourly blood glucose levels for many days, usually weeks, until optimal treatment can be safely established to enable a baby to be discharged home without the risk of hypoglycemia (5). These babies are usually managed without the added risks of central lines, but require repeated blood sampling from heel pricks or venipunctures that are distressing for the babies and demanding of resources.

Continuous glucose monitoring (CGM) is now used widely in adults and children with type 1 diabetes mellitus to reduce the risk of hypoglycemia (6); however, CGM in neonates remains limited despite the risks of glucose instability in newborns. Several CGM devices are available for clinical use that provide glucose levels in real time, with the leading manufacturers being Medtronic (Northridge, CA, USA) and Dexcom (San Diego, CA, USA). Despite significant advances in the technology, current devices are not designed or licensed for use in babies, and there are concerns about their accuracy in assessing hypoglycemia (7).

Previous neonatal studies have used masked CGM to characterize glucose control in newborns at risk of transient hypoglycemia (8). These studies have shown clinically silent episodes of hypoglycemia to be associated with worse visuomotor and executive function in early childhood (7). Other studies have demonstrated that CGM can reduce the risk of hyperglycemia and hypoglycemia in preterm infants (9, 10). Masked CGM has been used in children with CHI at home once treatment has been established, and similarly highlighted clinically silent hypoglycemia (11). However, the potential use of real-time CGM in newborns with CHI or persistent hypoglycemia because of prematurity has not been described (9). Having successfully used the CGM in our preterm population as an adjunct to support the targeting of glucose control in the perinatal period, we extended its use to infants with persistent hypoglycemia. The aim was to better understand the patterns of dysglycemia and reduce the risk of hypoglycemia and the need for such frequent blood glucose measurements.

## Materials and Methods

This is the report of the single-center use of real-time CGM for infants with a clinical diagnosis of persistent hypoglycemia. It reports on data collected over a 4-year period, using Medtronic Paradigm and Dexcom G4 systems. Persistent hypoglycemia was defined as recurrent hypoglycemia for > 48 hours (12). CGM sensor insertion, removal, and data uploading were undertaken by a specifically trained clinical team. Training was provided to the neonatal nursing team regarding calibration and use of real-time CGM to guide the need for blood glucose (BG) sampling dependent on the CGM absolute value and trends. Clinical data were collected retrospectively from the electronic medical records, along with all time-stamped point-of-care BG measurements. BG analyses were undertaken using the Novostat (Nova Biomedical, Waltham, MA, USA) glucose meter or a blood gas analyzer.

Standard neonatal clinical guidelines aimed to maintain BG levels in the range of 2.6 to 10 mmol/L, with the lower threshold rising to > 3.5 mmol/L in infants diagnosed with hyperinsulinism. A single BG < 2.6 mmol/L would warrant clinical review and treatment with a bolus of 2 mL/kg 10% dextrose along with review of current nutritional strategy as well as ensuring there were no problems with IV access. If at this time babies were on IV fluids, then the dextrose load would be increased and if infants were being fed enterally, frequency of feeds would be increased or additional IV dextrose would be started. If BG levels rose to > 10 mmol/L then an infant would be reviewed clinically to determine if there was any underlying etiology requiring treatment such as infection as well as calculation of dextrose load to ensure this was not excessive.

The real-time CGM devices have hypoglycemia alarms for trends and absolute glucose levels. However, the persistent nature of these alarms, even after clinical interventions, was identified early by nursing staff to be unhelpful, and therefore the alarms were turned off, but nurses were tasked to check sensor glucose levels hourly and respond to trends in readings. The device’s potential use to support clinical care was explained to the parents, and verbal consent was obtained.

### Insertion Technique for CGM Device in the Newborn

**Figure 1.**
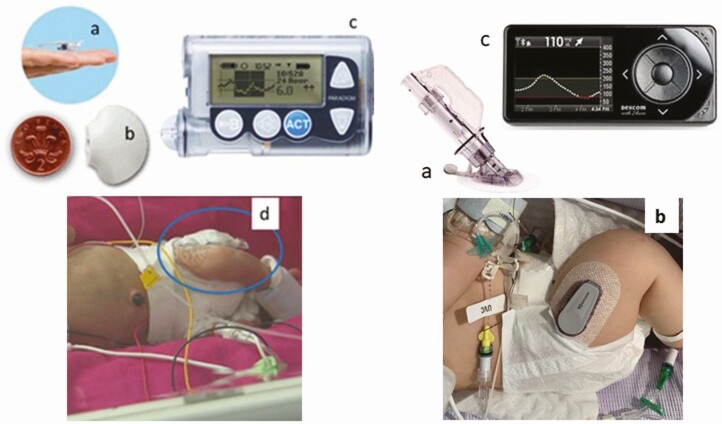
Infants with continuous glucose monitoring in situ. Left panel showing Medtronic Paradigm in a preterm baby: (A) sensor, (B) transmitter, (C) monitor, (D) baby with sensor in thigh. Right panel showing a Dexcom G4 with: (A) needle inserter, (B) transmitter on a baby’s thigh, and (C) monitor. Adapted with permission from Thomson* et al*. (13).

Two different real-time CGM devices were used and each required a specific insertion technique (Fig. 1). The Medtronic system was used in smaller preterm babies because the Enlite sensor is shorter and finer than that in the Dexcom system. However, because of the limited subcutaneous tissue in preterm babies and therefore the impracticality of a 90° angle of insertion (which is the case when using the Medtronic standard manufacturer’s inserter), these sensors were inserted without using the inserter provided. This technique has previously been described (14). In contrast, the Dexcom sensor insertion is angled at 45°, and this system was used in larger babies and inserted per the manufacturer’s instructions.

## Data Handling and Analyses

At the end of each monitoring period, the CGM data were uploaded to a secure database by a specifically trained nurse specialist. Patterns of dysglycemia were compared between preterm infants and those with CHI. This included calculating the coefficient of variation (CV) for each baby throughout the study period. The rapid fluctuation in CGM was defined as a change of > 10 mmol/hour (15).

Prespecified comparative analyses were undertaken between BG levels and CGM values, with real-time CGM data compared with the following BG level that were recorded within a 5-minute window. If duplicate BG levels were recorded within this 5-minute window, the single measurement closest to the time of CGM reading was selected to avoid bias to samples that had been repeated. We calculated the mean absolute relative difference as the mean percentage difference between the 2 measures (16). Bland-Altman analysis was used to assess the systematic differences between glucose measurements and Clarke error grid plots were used to explore the potential clinical impact. The Clarke error grid separates the scatter plot of data into 5 zones of clinical significance (17). The zones are defined according to the presence of error in the accuracy of the measurement combined with the severity of the consequence of subsequent treatment error. If a new method has a percentage (>95%) in zones A and B, it is considered clinically acceptable (17).

To assess clinical utility, the sensitivity and specificity of the real-time CGM in detecting an episode of hypoglycemia (BG < 2.6 mmol/L) was calculated. A clinical episode of hypoglycemia was defined as a BG < 2.6 mmol/L separated from another BG value < 2.6 mmol/L by more than 15 minutes. A CGM episode of hypoglycemia was defined as a single or continuous period of CGM values < 2.6 mmol/L separated from another episode of CGM value(s) < 2.6 mmol/L by > 15minutes. CGM episodes of hypoglycemia were categorized into 4 groups according to the duration of the hypoglycemia: < 15 minutes; 15 to 30 minutes; 30 to 60 minutes; and > 60 minutes. In keeping with previous literature, we considered continuous episodes of CGM < 2.6 mmol/L lasting > 60 minutes to be clinically significant. Adverse device effects were defined as an impact on skin integrity and signs of local infection

## Results

Fourteen neonates with persistent hypoglycemia were managed with the support of real-time CGM. These babies fell into 2 main subgroups: term babies with classical hyperinsulinism (n = 7), all of whom were treated and responsive to diazoxide; and preterm infants (n = 6) with persistent hypoglycemia with or without hyperinsulinism (Table 1). In addition, 1 term infant, whose sibling had classical hyperinsulinism but who had relatively short period of hypoglycemia, was included in the study. Seven babies had Medtronic sensors inserted and 7 babies had Dexcom sensors inserted. Sensors were inserted on median 14 days of age (range, 2-98), with a median duration of 13.7 (2.85-54.74) days. A total of 224 days of real-time CGM data were collected, with a median of 5.7 (3-10.4) BG tests per patient per day. There were 1254 paired CGM and BG values for comparison.

**Table 1.** Demographic details of babies managed using CGM

Gestational age (weeks + days)	Birthweight (g)	Birthweight SD score	Age at start of CGM (days)	Length of CGM use (days)	Diagnosis	Day diazoxide started	CGM system
39 + 2	2740	-1.5	27	4.2	CHI	16	Medtronic
38	2990	-0.4	4	30.1	CHI	7	Dexcom
37 + 1	3830	+1.8	5	22.7	Beckwith Wiedemann Syndrome	14	Dexcom
37 + 1	3978	+2.4	10	21.9	CHI - HNF4A	11	Dexcom
37 + 5	2100	-2.3	42	5.18	CHI, IUGR	7	Dexcom
37 + 2	3375	+0.78	4	16.85	CHI	18	Dexcom
37	2680	-0.3	6	9.48	Beckwith Wiedemann syndrome	8	Dexcom
36	1700	-2.4	2	17.5	CHI, IUGR	21	Dexcom
30 + 6	800	-2.75	18	54.74	CHI, IUGR	43	Medtronic
28	772	-1.8	50	11.6	IUGR	NA	Medtronic
27 + 4	420	-3	59	5.97	IUGR	NA	Medtronic
27 + 2	1015	+0.0	19	4.8	CHI	NA	Medtronic
27 + 6	680	-1.9	98	15.8	CHI, IUGR	88	Medtronic

Abbreviations: CGM, continuous glucose monitor; CHI, congenital hyperinsulinism; HNF4A, hepatocyte nuclear factor-4-alpha; IUGR, intrauterine growth restriction.

### Glucose Profiles on Real-time CGM

**Figure 2.**
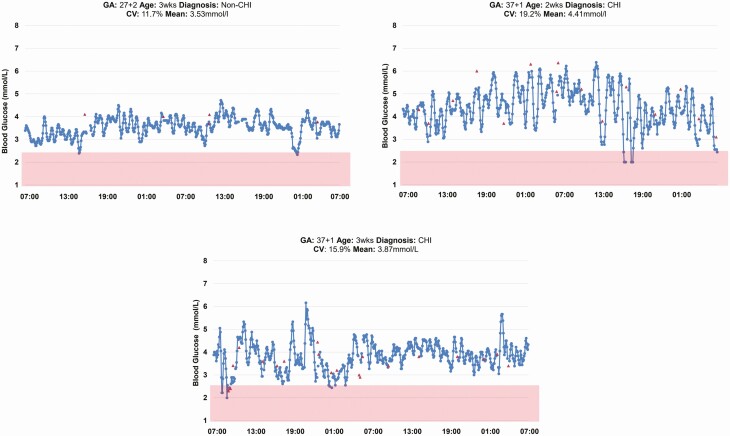
Comparison of continuous glucose monitoring patterns of a preterm baby with 2 term babies with hyperinsulinism. All babies were on full hourly enteral feeds and the term babies were both on diazoxide. Patterns are suggestive of more rapid fluctuations in glucose levels in the infants with congenital hyperinsulinism compared with the preterm infant. CHI, congenital hyperinsulinism.

**Figure 3.**
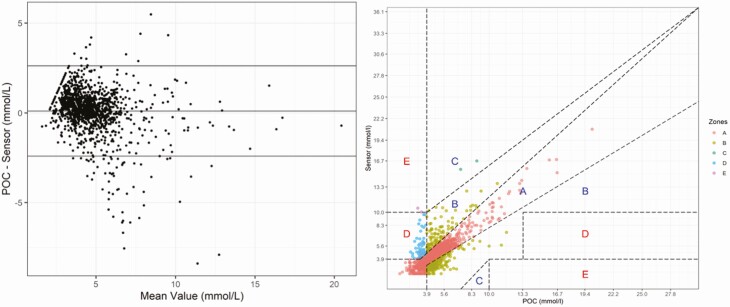
Comparison of continuous glucose monitoring (CGM) and point of care (POC) blood glucose levels. (A) Bland Altman plot. Mean represent mean of POC blood glucose and paired CGM sensor glucose levels. (B) Clarke error grid: data points in each zone: A981 (75%); B266 (20.6%); C2 (0.2%;) D56 (4%); E2 (0.2%).

Representative CGM patterns of infants on full hourly enteral feeds, comparing a term infant with hyperinsulinism (on diazoxide) with a preterm infant are shown in (Fig. 2). We observed considerable variations in the CGM glucose levels over very short time periods with mean (SD) of the difference in variation of 1.25 ± 0.74 mmol/L (range, 0.4-3.4 mmol/L) and the mean (SD) of the CV of 25.4 ± 10.4%. Exploring the CGM data showed that neonates with CHI tended to have greater SD (1.52 ± 0.79 mmol/L vs 0.77 ± 0.22 mmol/L, *P* = 0.07) and CV (29.3 ± 10.4% vs 18.3 ± 5.6%, *P* = 0.06) compared with the preterm infants, whereas the mean glucose levels were similar (5.10 ± 0.92 mmol/L vs 4.28 ± 0.80 mmol/L, *P* = 0.21). The CGM documented a clear rise in glucose levels after feeds and an increase in baseline glucose levels after diazoxide had been started, although rapid fluctuations remained characteristic of these traces (Fig. 3).

### Comparison of CGM and BG Levels

**Figure 4.**
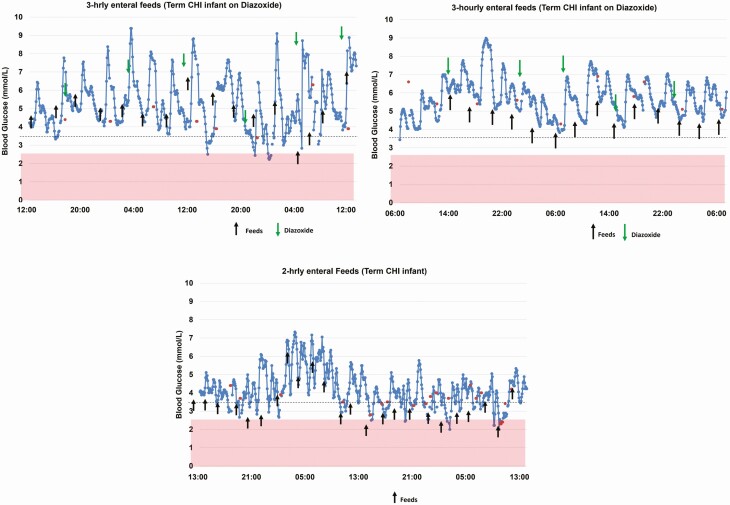
Continuous glucose monitoring pattern of term hyperinsulinemic babies in relation to frequency of enteral feeds. The rise in glucose levels in response to feeds at varying frequencies is demonstrated. POC, point of care.

Comparison of 1254 paired CGM and BG values are shown in the Bland Altman and error grid plots (Fig. 4A and 4B, respectively). There was no systematic bias in the difference between CGM and BG levels; the mean absolute relative difference was 11.0%. The Clarke error grid shows that 75% and 20.6% of the readings are in zones A and B, respectively, with CGM meeting the established criteria that > 95% of readings lie in either zone A or B (17).

## Clinical Hypoglycemia

In the 14 babies, over the study period of 224 days, there were a total of 22 episodes of “clinical hypoglycemia” (BG < 2.6 mmol/L). However, in none of these cases did the infants exhibit any clinical signs of hypoglycemia, but remained well throughout. In 13 episodes, the paired CGM glucose value was also < 2.6 mmol/L but in 9 of these episodes, the paired value did not match the BG threshold of < 2.6 mmol/L. Based on these data, the real-time CGM device had a sensitivity of 0.59, a specificity of 0.94, a positive predictive value of 0.15, and a negative predictive value of 0.99 to detect hypoglycemia. However, further interrogation of these data revealed that in 3 of these episodes, where the real-time CGM values were incongruent with the paired BG values, the real-time CGM sensor had previously reported a glucose level of < 2.6 mmol/L, and hypoglycemia treatment had been given before the BG level had been measured. Exclusion of these data in the analysis resulted in a calculation of the sensitivity in detecting hypoglycemia to 0.73 with no impact on specificity. It was also noted that episodes where the CGM did not document a glucose level < 2.6 mmol/L were associated with times when the glucose levels were falling rapidly (>10 mmol/L/h).

### Patterns of CGM Hypoglycemia

Our study shows that there were frequent episodes in some babies when the CGM would be transiently < 2.6 mmol/L, but that this was often for periods of < 30 minutes and was variable between babies (Fig. 2). Some of these instances will not have been noted clinically because the alarms were switched off and nurses only check CGM every hour. On other occasions because these “sensor glucose (SG) events” were fleeting and if recent BG has been normal and a baby was well, such brief transient dips were tolerated by the nurses without intervention. We reviewed the 13 episodes of more prolonged CGM hypoglycemia (CGM < 2.6 mmol/L, for > 60 minutes) in more detail. Of these 13 episodes, 2 (16%) were true hypoglycemia and were treated with IV bolus or oral glucose, but 11 were associated with normal BG (>2.6 mmol/L) with 7/11 (64%) having occurred within the first 24 hours of CGM insertion (18).

### Adverse Effects

There were no reported concerns regarding skin integrity or infection at the insertion site in any babies in our study. No babies showed any signs of clinical compromise or clinical seizures related to hypoglycemia during the study period.

## Discussion

This is the first report of real-time CGM as an adjunct to intermittent BG management in a cohort of neonates with persistent hypoglycemia and CHI. It demonstrates the value of CGM in revealing the rapid fluctuations in glucose levels in these babies, which makes clinical management and prevention of hypoglycemia challenging when using intermittent BG values.

The marked fluctuations in glucose levels in these babies not only makes clinical management difficult, but also creates challenges in validation of the accuracy of the CGM. Infants with CHI treated with diazoxide in the study showed higher variation in glucose levels measured by SD compared with the previously reported values in healthy term babies > 96 hours old (SD 1.52 and 0.70 mmol/L, respectively) (19). The CVs in some of the neonates were in the ranges reported in patients with type 1 diabetes with suboptimal metabolic control (> 30%) (20). In contrast, the variation in glucose levels in the preterm neonates without hyperinsulinism in the study was similar to reported in healthy term babies (SD 0.77 vs 0.70 mmol/L) (19).

These rapid fluctuations are relevant when using CGM because CGM measures interstitial fluid glucose levels, and there is a well-documented physiological lag between interstitial and BG levels (21). These differences in tissue compartments are known to be most significant with rapidly changing glucose concentrations (22). The large and rapid fluctuations in these infants’ glucose levels may have contributed to the high number of false-positive CGM hypoglycemic readings, with limitations in point accuracy. Previous studies in children with CHI have shown that CGM tends to under read compared with BG measurements; our data are in keeping with this (9). These data, combined with the better specificity for hypoglycemia, mean CGM is best placed to act as an adjunct on glucose trends and the timely need for intermittent BG monitoring during normoglycemia rather than point accuracy. The use of CGM to provide reassurance during periods of normoglycemia could potentially limit the need for such frequent blood sampling. The real-time data providing continuous trends that would highlight falling glucose levels and alert the clinician to the need for BG measurement rather than levels simply being taken hourly as part of routine care.

The study also highlighted a large number of short episodes with real-time CGM levels < 2.6 mmol/L, lasting for relatively short periods, and resolving without apparent intervention (Fig. 2). The real-time CGM data demonstrated that 94% of the total CGM hypoglycemic episodes (220 of 233 episodes), lasted less than an hour. Because of these episodes’ transient nature, a BG value was not always available for validation, and without CGM, they may well not have been detected by standard intermittent hourly BG monitoring. The clinical significance of these episodes remains to be determined. However, data collected using blinded CGM in infants at risk of transient neonatal hypoglycemia have demonstrated an association between clinically silent hypoglycemia, detected using CGM, and impaired executive and visuomotor outcomes at 4.5 years (23). Thus, CGM may well be a valuable tool not just to support avoidance of hypoglycemia, but also to help to determine the characteristics of hypoglycemic exposure, beyond simple thresholds (including length of exposure and effect of fluctuations) that affect longer term outcomes (24).

One of these data’s limitation is that this is a small single-center pragmatic report of real-time CGM use. The clinical data collection was dependent on the retrospective review of clinical notes. The lack of paired BG levels for all the transient episodes when the CGM < 2.6 mmol/L means the accuracy of these transient falls in CGM levels is unknown. The study is limited by the absence of a control group of babies managed according to “standard practice” to compare hypoglycemic exposure or BG sampling frequency. However, given the small numbers of babies with diverse pathologies, this was not believed to be practical. Furthermore, the frequency of hypoglycemic episodes in these babies is in keeping with the reported prevalence in the literature for infants with CHI (9). We did not undertake any formal assessment of the staff’s perception of utility, and this would be important to determine impact on clinical care. The study was undertaken at a tertiary neonate unit, not a specialist unit for hyperinsulinism. Further prospective studies would be warranted to review the accuracy and the use of CGM in these potentially higher risk infants.

Although CHI is considered a rare condition, increasing numbers of babies are being diagnosed and managed outside supra-specialist centers. This study demonstrates CGM is a potential strategy to support management in such centers; however, neonatal nurses typically have less CGM technology experience, and staff training would be critical to ensure that CGM was used appropriately as an adjunct to management alongside BG monitoring. Newer CGMs have improved accuracy and no longer require calibration with BG levels but further developments in the technology to optimize use in the newborn would be beneficial.

This study clearly highlights the challenges of maintaining normoglycemia in babies with CHI. The management of individual patients is demanding of staff time and can result in prolonged hospital admissions. Hourly BG sampling is stressful for infants; adjuncts such as real-time CGM may help improve characterization of patterns of glucose control and reduce the risk of hypoglycemia, encouraging more proactive management and affecting reduction in the length of neonatal stay (10, 25, 26).

## Data Availability

Requests for access to anonymized continuous glucose monitoring data should be made in writing to K.B.

## Abbreviations

BG: blood glucose
CGM: continuous glucose monitor
CHI: congenital hyperinsulinism
CV: coefficient of variation
GA: gestational age
